# The African Swine Fever Isolate ASFV-Kenya-IX-1033 Is Highly Virulent and Stable after Propagation in the Wild Boar Cell Line WSL

**DOI:** 10.3390/v14091912

**Published:** 2022-08-29

**Authors:** Johanneke D. Hemmink, Hussein M. Abkallo, Sonal P. Henson, Emmanuel M. Khazalwa, Bernard Oduor, Anna Lacasta, Edward Okoth, Victor Riitho, Walter Fuchs, Richard P. Bishop, Lucilla Steinaa

**Affiliations:** 1 Animal and Human Heath Program, International Livestock Research Institute, Nairobi 00100, Kenya; 2 Institute of Molecular Virology and Cell Biology, Friedrich-Loeffler-Institut, 17493 Greifswald-Insel Riems, Germany; 3 International Livestock Research Institute, Nairobi 00100, Kenya

**Keywords:** African swine fever virus, genotype IX, Kenyan isolate, virulence

## Abstract

We describe the characterization of an African swine fever genotype IX virus (ASFV-Kenya-IX-1033), which was isolated from a domestic pig in western Kenya during a reported outbreak. This includes the efficiency of virus replication and in vivo virulence, together with genome stability and virulence, following passage in blood macrophages and in a wild boar lung cell line (WSL). The ASFV-Kenya-IX-1033 stock retained its ability to replicate in primary macrophages and retained virulence in vivo, following more than 20 passages in a WSL. At the whole genome level, a few single-nucleotide differences were observed between the macrophage and WSL-propagated viruses. Thus, we propose that the WSL is suitable for the production of live-attenuated ASFV vaccine candidates based on the modification of this wild-type isolate. The genome sequences for ASFV-Kenya-IX-1033 propagated in macrophages and in WSL cells were submitted to GenBank, and a challenge model based on the isolate was developed. This will aid the development of vaccines against the genotype IX ASFV circulating in eastern and central Africa.

## 1. Introduction

African swine fever (ASF) is a hemorrhagic disease of pigs, which causes up to 100% mortality in naïve domestic pigs. Although African swine fever virus (ASFV) has been endemic in sub-Saharan Africa (SSA) for decades, other parts of the world are also affected. In SSA, multiple genotypes, based on the variable c-terminal sequence of the gene encoding the p72 major surface antigen [1], circulate simultaneously, e.g., five genotypes were identified in Tanzania and neighboring countries between 2005 and 2018 (Reviewed by [2]). Genotypes IX and X are most common in eastern Africa, but genotypes II and XV have also been detected in Tanzania [3,4,5,6]. Following the introduction of the genotype I ASFV to Europe in the 1950’s and its subsequent eradication (except for Sardinia), a genotype II ASFV was introduced to the Caucasus from Southeast Africa in 2007 and has subsequently disseminated widely to Russia, eastern Europe, East Asia, including China, and most recently, Latin America [7,8]. In the absence of globally available treatments or commercial vaccines, ASFV represents a serious threat to the global pig industry.

Efforts are being made to develop vaccines against this devastating virus, with most efforts focused on the genotype II ASFV, which is currently circulating in Europe, Asia, and Hispaniola. The most promising candidate vaccines are live-attenuated African swine fever viruses (LA-ASFV), which have shown to provide up to 100% protection against challenges from homologous pathogenic ASFVs [9,10,11,12,13]. The live-attenuated viruses show reduced virulence, due to the deletion of genes associated with virulence, either naturally [9] by passage in tissue culture [14,15] or by genetic modification techniques [16,17,18,19].

One of the challenges with the development of LA-ASFV is the difficulty of growing ASFV in vitro. ASFV is normally grown in primary cells, such as blood-derived macrophages or pulmonary alveolar macrophages. However, the use of a continuously growing production cell line is desirable, since this allows improved quality control. However, prolonged passage in tissue culture can alter the ASFV genome, the in vitro characteristics of the virus, and/or the in vivo characteristics [14,15,20].

The wild boar cell line (WSL) is isolated from lung tissue and grows continuously without the addition of any propagating factors. It was originally described as a pulmonary macrophage line [21], but a later proteomic study didn’t support this finding, suggesting that the WSL belongs to the fibroblast lineage [22]. It appears that the WSL has a solid and broad antiviral innate immune response to transfection with non-coding RNAs [23]. The cell line has been reported to support the growth of ASFV [19,24,25], but limited information is available on whether propagation of ASFV in the WSL alters the genome and/or the in vivo characteristics of the virus, as seen after propagation in other cell lines, such as in Vero cells [14]. To elucidate how propagation in the WSL affects a genotype IX ASFV isolated from a domestic pig in western Kenya (ASFV-Kenya-IX-1033) [26], we investigated the in vitro growth, in vivo virulence, and genomic stability of ASFV-Kenya-IX-1033 stocks propagated in either blood-derived macrophages or WSL [26].

## 2. Materials and Methods

### 2.1. Expansion of Viral Stocks

ASFV-Kenya-IX-1033 was isolated from the spleen of an infected domestic pig from the Busia district in western Kenya [4]. The isolated virus was passaged twice in blood macrophages before its adaptation to the wild boar cell line (WSL) at the Friedrich-Loeffler-Institute (FLI), where it underwent ~20 passages (p20) [21,25].

The WSL-grown virus (p20) was transferred back to the International Livestock Research Institute (ILRI) in Nairobi, Kenya, where it underwent two additional passages in WSL. The WSLs were kindly provided by Matthias Lenk from the Friedrich-Loeffler-Institute (FLI), Greifswald-Insel Riems, Germany. It is not commercially available. For each passage, WSL cells were infected at 80% confluence with a multiplicity of infection (MOI) of 0.1, in either T25 or T75 flasks, and incubated at 37 °C and 5% CO_2_ for 5–7 days. Cells and supernatant were harvested from flasks, and cells were lysed by repeated freeze-thawing three times. The virus-containing supernatant was clarified by centrifugation at 670× *g* for 10 min and aliquoted and stored at −80 °C. Stocks were titrated using HAD_50_ assay in pulmonary alveolar macrophage (PAM) cells, isolated as described by Zhang et al. [27], or by TCID_50_ assay in WSL cells.

The macrophage-grown virus stock of ASFV-Kenya-IX-1033 underwent a total of four passages in blood macrophages. For each passage, blood macrophages were infected at MOI 0.1, in either T25 or T75 flasks, and were incubated at 37 °C and 5% CO_2_ for 5–7 days. Cells and supernatant were harvested from the flasks, and cells were lysed by repeated freeze-thawing three times. The virus-rich supernatant was clarified by centrifugation at 670× *g* for 10 min, and the clarified supernatant was aliquoted and stored at −80 °C. Stocks were titrated using HAD_50_ assay in PAM.

### 2.2. Purification of African Swine Fever Virus

For whole-genome sequencing, the clarified supernatants containing ASFV, for both the WSL-grown and the blood macrophage-grown virus stocks as described in the previous section, were further purified using a 36% sucrose solution. The supernatant was transferred into autoclaved, 250 mL, flat-bottom ultracentrifuge tubes, followed by centrifugation at 18,500× *g* for 2 h at 4 °C to pellet the virus particles in a Beckman Coulter Avanti Centrifuge J-301. The pellet was resuspended in 3 mL of 10 mM Tris (pH9), and the virus suspension was layered on 36% sucrose solution and subjected to ultracentrifugation at 30,000× *g* for 2 h at 4 °C, using the Beckman Coulter Optima XE-90 ultracentrifuge. The pellet containing the purified virus was resuspended in 10 mM Tris (pH9) and aliquots were stored at −80 °C.

### 2.3. Whole-Genome Sequencing, Genome Assembly and Sanger Sequencing

DNA was extracted from the sucrose-purified virus using the Qiagen DNeasy blood and tissue kit (Qiagen, UK), according to the manufacturer’s protocol. Whole-genome sequencing was performed using the Illumina MiSeq platform at ILRI, as described previously [19]. Reads were trimmed to remove low-confidence bases using Trimmomatic (release 0.38, [28]), with the following parameter settings: LEADING: 10; TRAILING: 10; SLIDINGWINDOW: 4:20; and MINLEN: 25. Host reads were eliminated by mapping the trimmed reads to the Sus scrofa genome (assembly 11.1) using the Bowtie 2 aligner (v2.3.4.1, [29]). De novo assembly was generated using Unicycler (v0.4.7, [30]), which uses the SPAdes assembler to generate de novo assemblies. The assembled contigs were annotated against Ken06.Bus, GenBank accession: KM111295 [3], using RATT v1.0.3 [31], with the strain preset parameters. Annotated genes were manually checked. Further improvement to the automated annotation was carried out; additional open reading frames were identified and annotated as putative genes if their homologues were present in published genomes; alternative transcripts were identified based on data published in Cackett et al., 2020 [32].

To verify SNPs and indels in the whole genome sequence, loci of interest were amplified, with respective primer pairs (Appendix A Table A1). The resulting amplicons were purified using the High Pure PCR product purification kit (Roche) and shipped to Macrogen Europe B.V. (Amsterdam, The Netherlands) for Sanger sequencing with the same primers. The sequences were then analyzed using SnapGene (GSL Biotech, Chicago, IL, USA).

### 2.4. Growth Kinetics

WSL cells and PAMs were infected with either the blood macrophage- or the WSL-grown virus stocks at different multiplicities of infection (MOI) and incubated at 37 °C and 5% CO_2_ in duplicate wells of a 24-well plate. After 2 h of incubation, cells were washed twice in 1× PBS to remove non-attached and non-internalized viruses, before the addition of the complete medium (RPMI 1640 for PAM (Sigma Aldrich, Gillingham, UK) or DMEM for WSL-(Sigma Aldrich, UK), supplemented with 2 mM L-glutamine (Sigma Aldrich, UK), 10% fetal bovine serum (FBS), 100 UI/mL penicillin (Sigma Aldrich), and 100 mg/mL streptomycin (Sigma Aldrich, UK)). Cells and supernatant were harvested at 2, 24, 48, 72, and 96 h after infection and frozen at −80 °C until further analysis. After 3 freeze–thaw cycles, viral titers were established using HAD_50_ assay using PAMs. Viral titers in the supernatant were measured on the same day for consistency in results.

### 2.5. In Vivo Experiments

All animal experimental work was approved by the ILRI Institutional Animal Care and Usage Committee (IACUC2019-05, IACUC2020-11 and IACUC2020-18). Pigs did not have detectable ASFV DNA copies in EDTA blood by qPCR and were seronegative, as determined by a competitive p72 ELISA (Ingezim PP3 COMPAC, Ingenesa, Spain) prior to the start of the experiment. Groups of 5 animals were inoculated by intramuscular injection in the neck with 1 or 10^2^ HAD_50_ of the blood macrophage-propagated virus stock or 10^2^ TCID_50_ of the WSL-propagated virus stock. Infected animals were monitored daily, and clinical scoring was performed daily, according to King et al. [9]. Pigs were euthanized using a barbiturate overdose after sedation with ketamine and xylazine when the humane endpoint criteria were reached. Serum, EDTA blood samples, and nasal swabs were taken on days 0, 3, 5, and 7 post infection. Serum samples were used for the determination of viremia. Tissue samples were obtained during post-mortem investigation for the determination of viral titers in tissue.

### 2.6. Determination of Viral Titers by HAD_50_

Virus titers of viral stocks and experimental samples were determined by HAD_50_ assay. The virus containing samples were 10-fold serial diluted in complete RPMI (described above) and added to PAM in 96-well plates, in 4 replicates per dilution. The presence of the virus was assessed by hemadsorption at day 5, after the addition of red blood cells and viral titers were calculated by the Spearman–Kärber algorithm, as described/reviewed in Ramakrishnan et al. [33].

### 2.7. Determination of Viral Titers by p72/B646L qPCR

p72/B646L qPCR was used to assess the virus DNA content in tissue samples. DNA was extracted from tissue using the Qiagen DNeasy blood and tissue kit (Qiagen, UK), according to the manufacturer’s protocol. qPCR was performed as per the OIE-recommended real-time PCR assay, according to King et al. (2003) [9], but primer and probe sequences were adapted to genotype IX. Primer sequences were P72-F ‘CTGCTCACGGTATCAATCTTATCGA’, P72-R ‘GATACCACAAGATCAGCCGT’, and P72 probe ’FAM-CCACGGGAGGAATACCAACCCAGCG-TAMRA3′.

### 2.8. Statistical Analyses

The growth kinetics of the blood macrophage-grown and WSL-grown viruses were compared using a two-way ANOVA for growth in macrophages and for growth in WSL. A comparison of their survival was tested using a Mantel-Cox log rank test. A two-way ANOVA was used to analyze the viral growth kinetics. The time to first detection of virus in serum, the time to maximum viral titer in serum, the time to humane endpoint, and the CT values in tissues were analyzed using a one-way ANOVA. All statistical analyses were performed using Graphpad Prism version 8, (GraphPad Software, Inc., La Jolla, CA, USA).

## 3. Results

### 3.1. In Vitro Growth of Blood Macrophage Propagated and WSL Propagated ASFV-Kenya-IX-1033

**Figure 1:**
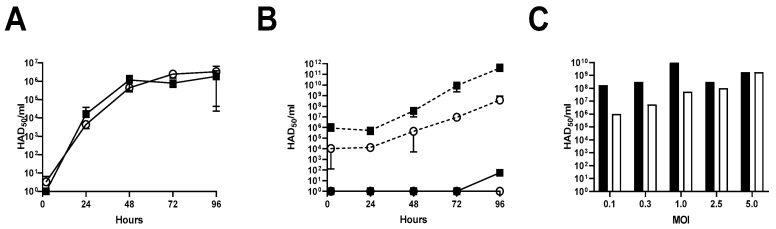
Viral growth kinetics of WSL-propagated (▪) or blood macrophage-propagated (○) ASFV-Kenya-IX-1033 in WSL and PAM. PAM (**A**) or WSL cells (**B**) were infected with MOI 0.01 (continuous line) or MOI 1 (dashed line) of the macrophage-propagated (**○**) or the WSL-propagated (**▪**) virus stocks. Washing was performed after 2 h, and samples were collected at different timepoints (0/2, 24, 48, 72, and 96 h post infection). Viral titers were determined in HAD_50_ assay. (**C**) WSL cells were infected with different MOIs of the macrophage-propagated (open bars) or the WSL-propagated (black bars) virus stock for 4 days in single determinations. No washing step was performed, and samples were collected at 96 h post infection.

Viral growth kinetics were determined in both the PAM and WSL to assess the efficiency of infection and expansion of the different viral stocks (blood macrophage-grown and WSL-grown viruses). PAMs were infected with the two virus stocks at a MOI of 0.01. Similar growth (no statistical difference using a two-way ANOVA) was seen for both the macrophage-grown stock and the WSL-grown virus in the PAM, with final titers of approximately 5 × 10^6^ HAD_50_/mL for the two stocks after 96 h (Figure 1A) (group effect, *p* = 0.4152; time effect, *p* = 0.0667, interaction 0.6189). Growth in WSL cells was less efficient for both the blood macrophage- and WSL-propagated virus stocks; the virus was only detected after 96 h when using an MOI 0.01 with a wash step after 2 h. However, when the MOI 1 was used (Figure 1B), an increase in viral titer was observed from 48 h post infection, with lower viral titers observed for the blood macrophage-propagated stock compared to the WSL-propagated stock (3.8 × 10^8^ HAD_50_/mL vs. 3.9 × 10^11^ HAD_50_/mL), albeit this was not statistically different (group effect, *p* = 0.2797; time effect, *p* = 0.3541; interaction, *p* = 0.3558). When testing the viral titers after 4 days of in vitro culture using different a MOI of the virus, without a wash step after infection, we observed the same tendency as seen in Figure 1B, with lower titers observed for the blood macrophage-propagated stock compared to the WSL-propagated stock, depending on the MOI used; at MOI 0.1, the titers were 1 × 10^6^ HAD_50_/_mL_ and 1.7 × 10^8^ HAD_50_/mL for the blood macrophage-propagated and the WSL-propagated virus, whereas at MOI 5, both stocks had the same viral titer (1.7 × 10^9^ HAD_50_/mL) (Figure 1C).

### 3.2. Virulence of Blood Macrophage-Grown versus WSL-Grown ASFV-Kenya-IX-1033

**Figure 2:**
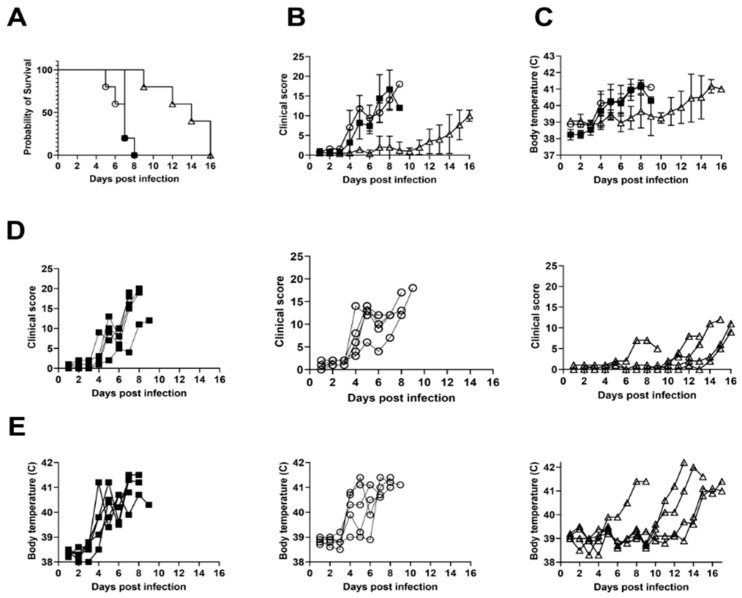
Clinical data from pigs inoculated with WSL-grown and macrophage-grown viruses. (**A**) The survival, (**B**) mean clinical scores, and (**C**) mean body temperatures after inoculation with 10^2^ TCID_50_ of WSL grown virus (▪), 10^2^ HAD_50_ blood macrophage grown virus (○), or 1 HAD_50_ blood macrophage grown virus (∆). (**D**) Individual clinical scoring data and (**E**) body temperatures for the animals in the different groups.

To investigate if the propagation of the virus in different cell types affected the virulence/pathogenicity of the virus in vivo, animals were infected by intramuscular injection with 1 HAD_50_ or 10^2^ HAD_50_ of the macrophage-propagated ASFV-Kenya-IX-1033 or 10^2^ TCID_50_ of the WSL-propagated ASFV-Kenya-IX-1033. Both the macrophage- and the WSL-propagated virus stocks were highly pathogenic in vivo. All animals developed severe clinical signs compatible with ASF. There was no statistical difference between the final survival of the groups inoculated with the high dose of either the blood macrophage- and the WSL-propagated ASFV-Kenya-IX-1033 viruses using a Mantel-Cox log-rank test (*p* = 0.395). However, a delay in the clinical outcome was observed in animals inoculated with a lower dose of 1 HAD_50_ of the blood macrophage-grown stock (Mantel-Cox log-rank test (*p* = 0.0017). The animals reached their humane endpoint between day 9 and day 16 after infection with 1 HAD_50_ (Figure 2) (*p* = 0.001 using one-way ANOVA).

Similarly, the clinical scores and body temperatures between the groups inoculated with the high dose of blood macrophage- and WSL-propagated ASFV-Kenya-IX-1033 were also similar, but a delay in the rise of the clinical score and the body temperature was observed for the group inoculated with the low dose of the blood macrophage-grown virus.

**Figure 3:**
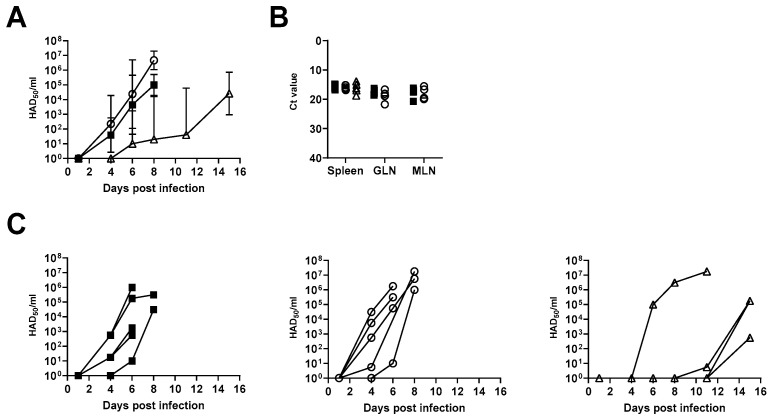
Viral titers in serum and tissues. (**A**) Mean virus titers (geometrical) ± SD (geometrical) in pig serum after inoculation with 10^2^ TCID_50_ of the WSL-grown virus (▪), 10^2^ HAD_50_ blood macrophage-grown virus (○), or 1 HAD_50_ blood macrophage-grown virus (∆). HAD_50_ titers in serum, the geometrical mean with the geometrical standard deviation, is displayed; (**B**) Ct values obtained by qPCR using DNA extracted from tissues obtained at postmortem from the spleen, gastro-hepatic lymph node (GLN), or mesenteric lymph node (MLN); (**C**) individual HAD_50_ titers in serum.

No difference was observed in time to first detection of the virus in the serum between animals inoculated with the higher doses of the blood macrophage- and the WSL-propagated ASFV-Kenya-IX-1033 (respectively, time to first virus detection is 6.00 ± 2.00 and 5.4 ± 0.896) (Figure 3A,C). However, a delay in the detection of the virus in the serum was observed in animals inoculated with the lower dose of 1 HAD_50_ of the blood macrophage-propagated stock (time to first virus detection, 11.800 ± 3.899, *p* = 0.002) (Figure 3A,C). Similarly, there was no difference in time to maximal titer between the two high-dose groups, but there was a delay in the group inoculated with 1 HAD_50_ of the blood macrophage-grown stock (5.8 ± 1.095, 6.00 ± 1.095 and 12.800 ± 1.789, *p* < 0.0001). There were no differences in maximal titers of the virus in the serum between the three groups (*p* = 0.45), and no differences were observed between the three groups in CT values obtained by p72-qPCR, using DNA from tissues harvested after euthanasia/death (*p* = 0.616) (Figure 3B).

**Figure 4:**
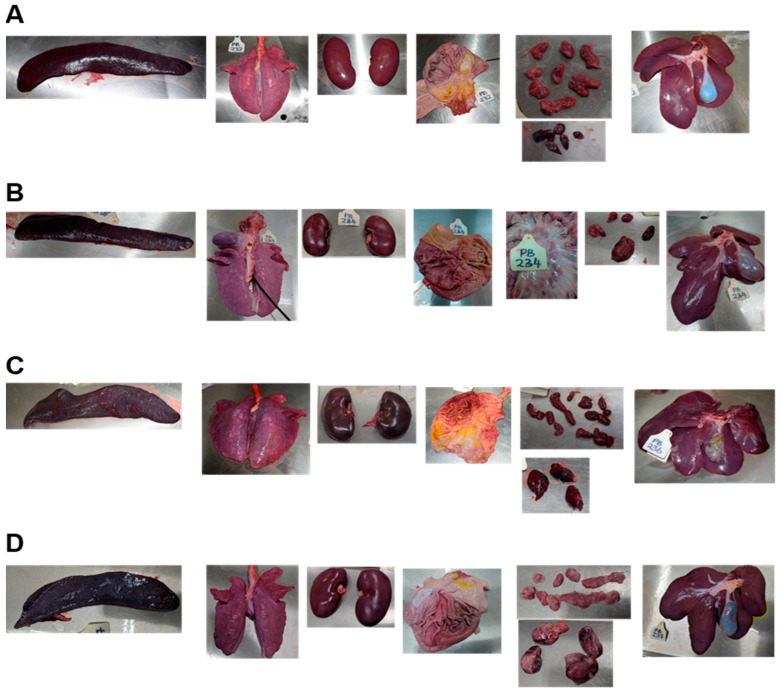
Gross pathology of ASFV-inoculated pigs. Organs from two pigs of each group are shown as examples. (**A**,**B**) Pigs were inoculated with 10^2^ TCID_50_ ASFV-Kenya-IX-1033, grown in WSL, or (**C**,**D**) 10^2^ HAD_50_ ASFV-Kenya- IX-1033, grown in blood macrophages. From left to right are displayed the spleen, lung, kidneys, stomach mucosa, mesenteric and gastro hepatic lymph node, and liver.

Gross pathological findings were compatible with acute African swine fever for animals inoculated with both the ASFV-Kenya-IX-1033 grown in the WSL and ASFV-Kenya-IX-1033 grown in blood macrophages (Figure 4). Findings included an enlarged hemorrhagic spleen; mild to severe lung edema, with, in some cases, hemorrhages throughout the lung tissue; dark hemorrhagic kidneys; and hemorrhages in the mucosa of the stomach and/or the esophagus. For some animals, hemorrhages were also seen in the mucosa elsewhere in the gastrointestinal tract. All lymph nodes were enlarged, and most were hemorrhagic or had a blood clot-like appearance, including the renal lymph nodes, gastrohepatic lymph nodes, and mesenteric lymph nodes.

### 3.3. Whole Genome Comparison of Blood Macrophage Grown versus WSL-Grown ASFV-Kenya-IX-1033

As there were limited in vitro and in vivo differences between the macrophage-grown and WSL-grown ASFV-Kenya-IX-1033 stocks, whole-genome sequencing was performed to establish the degree of genomic changes between the stocks. DNA from macrophage- and WSL-grown viruses was sequenced using the Illumina MiSeq platform, which yielded 2.9 M and 3.3 M paired-end reads, respectively. After the removal of short, low-confidence reads and those derived from the host, about 700,000 (13.3%) and 1.2 M (18.8%) reads remained, which were assembled de novo. Both the macrophage- and WSL-grown virus genomes assembled into two contigs, the total number of bases assembled being 182,424 bp and 182,038 bp, respectively. The two contigs were separated by a break of 293 bp and 678 bp in the N-terminal region of the CD2v gene in the two genomes, respectively. To check whether the contig break was due to a deletion in the virus DNA or an anomaly arising from the sequencing, we designed PCR primers flanking the putative breaks (Appendix A Table A1) and sequenced the resulting amplicons by using the Sanger method, confirming that there was no deletion in the virus genome at the CD2v locus.

A high level of nucleotide identity (>99%) between the two ASFV-Kenya-IX-1033 stocks was observed across the genome, with four single-nucleotide polymorphisms (SNPs) present in the aligned regions (Table 1). Of the four SNPs, one was in a polyG tract in the intergenic region between the MGF 360-7L and X69R genes. Three SNPs were in coding regions and two were non-synonymous mutations, resulting in alanine to threonine substitutions in the MGF 505-2R and D250R (g5R) genes. The other mutation was a synonymous mutation in the I329L (k11L) gene. The SNPs in the coding regions were confirmed by Sanger sequencing of PCR products targeting the regions of interest (Table 1).

The genomes of the two ASFV-Kenya-IX-1033 stocks were annotated based on the Ken06.Bus strain [3], which had 99% sequence similarity to the stocks. The Ken06.Bus strain has 161 annotated genes; 159 of the genes were present in the ASFV-Kenya-IX-1033 stocks. Genes annotated as MGF 110-11L (FRAG-2) and MGF 110-12L were absent in the ASFV-Kenya-IX-1033 stocks. In addition to the genes discovered by annotation transfer from Ken06.Bus, five additional coding sequences were identified in the genomes, based on sequence similarity with putative novel genes described in the reannotation of the genotype I strain, BA71V, which is currently the most comprehensively annotated ASFV genome [32]. Sequence data generated for the macrophage-grown and WSL-grown ASFV virus in this study were submitted to GenBank under SRA accessions SRR17226616 and SRR15187368 [19], respectively.

## 4. Discussion

In eastern Africa, multiple genotypes of ASFV are concurrently circulating, with genotype IX and X being responsible for most outbreaks [3,4,26]. Additional genotypes, including genotype II and XV, have been detected in Tanzania [5,6]. In this study, we describe the in vitro and in vivo characterization of ASFV-Kenya-IX-1033, which was isolated in Kenya near the border to Uganda. The availability of a well-defined strain of the genotype IX ASF virus, as described in this report, is key to testing candidate vaccines for the eastern African region, where this genotype, together with the very similar genotype X, are the predominant genotypes responsible for disease [34]. The strain is highly pathogenic in vivo, with 100% of experimentally infected animals reaching the predetermined humane endpoint criteria. At a dose of 10^2^ HAD_50,_ animals reached the humane endpoint criteria between 5 to 8 days post injection, and this was reproducible over several experiments. Even at a dose of 1 HAD_50,_ all animals reached the humane endpoint between 9 to 16 days post challenge.

There are several promising candidate vaccines based on the LA-ASFV, which show protection against homologous ASFV challenges, with up to 100% protection. One of the challenges with the development of LA-ASFV is the difficulty of growing ASFV in vitro. ASFV is traditionally grown in primary cells, such as blood-derived macrophages or PAMs. However, a continuously growing cell line for the propagation of ASFV would substantially reduce the cost for vaccine production and allow improved quality control. However, prolonged propagation in continuously growing cell lines can lead to genomic changes. Growth of ASFV in Vero cells, which is an immortalized primate cell line, resulted in large deletions in the ASFV genome as the virus adapted to the cell line [14]. In the case of the BA71 isolate, adaptation to Vero cells (BA71V) led to a non-virulent ASFV, which was associated with dramatic genomic changes between the BA71 and the BA71v stocks [15]. Similarly, the adaptation of attenuated ASF-G-Δ177L to growth in porcine epithelial cells, maintained at the US Plum Island Exotic Disease facility (PIPEC), led to deletions of genes in the left variable region of the genome, namely seven genes of the MGF300 and MGF360 family, and a fusion of MGF360-4L with MGF360-11L (ASF-G-Δ177L/ΔLVR). Further passages in PIPEC resulted in few point mutations in ORFs, with additional mutations observed outside ORFs. Thus, there is an ongoing effort to identify production cell lines, which support the replication of ASFV while retaining viral genomic integrity [35,36]. In the present study, it was demonstrated that the WSL supports the propagation of ASFV-Kenya-IX-1033, and that the virus maintains its genomic stability and infectivity to pigs. Few genomic changes were seen in ASFV-Kenya-IX-1033 grown in WSL, compared to ASFV-Kenya-IX-1033 grown in blood macrophages. Even after the development of candidate gene-deleted LA-ASFVs, which undergo additional passages to obtain pure clones, few genomic changes were observed, indicating that the genome of ASFV-Kenya-IX-1033 is indeed very stable in WSL [19]. These genomic changes could be non-functional, since mutations occur in pathogens as they replicate, or they could result in adaptation to the cell line used for the growth of the virus. To demonstrate whether the mutations are adaptative, repeated infection and genome analysis would be needed. Further investigation is needed to see whether other ASFV isolates can also be propagated in WSL while maintaining genomic stability. ASFV-Kenya-IX-1033 propagated in WSL was used as a backbone for the introduction of gene modifications using CRISPR/Cas9 technology for the development of candidate live-attenuated vaccines [19].

Both the blood macrophage- and the WSL propagated ASFV-Kenya1033-IX can infect WSL, but a tendency to lower viral titers were obtained for the blood macrophage-propagated virus compared to the WSL-propagated virus (not statistically different). We also observed lower final titer when a lower MOI was used, which simply may be related to a delayed replication. Viral titers of up to 3.9 × 10^11^ HAD_50_/mL were obtained using the WSL-propagated stock. Sanchez et al., found that ASFV viruses could grow in a PAM and WSL with lower titers at 12 h post infection in WSL cells for most viruses compared to PAMs, but similar viral titers were found for the viruses tested later, after in vitro infection (>48 h) (NHV/P68, Armenia/07 and E70) [24]. In this study we showed that after multiple passages in WSL, high viral titers can be obtained. Abkallo et al. demonstrated similar titers for ASFV-Kenya-IX-1033 and ASFV-Kenya-IX-1033 gene-deleted viruses (ASFV-Kenya-IX-1033-∆CD2v, ASFV-Kenya-IX-1033-∆A238L, and ASFV-Kenya-IX-1033-∆CD2v∆A238L), as determined by qPCR [19]. Therefore, the WSL cell may be beneficial for future application in commercial vaccine production, as many doses can be produced from a relatively small volume of culture.

Keßler et al. investigated differences in the intracellular ASFV proteome after the infection of different cell lines (WSL-HP, HEK293 or Vero) and found considerable quantitative differences in the expression of viral proteins, depending on the type of infected cells [37]. Deciphering the viral and host factors relevant for in vitro growth could allow for the targeting of genes for better in vitro growth.

In conclusion, the Kenyan ASFV isolate ASFV-Kenya-IX-1033 is a highly virulent virus, and this virulence is maintained after in vitro passage in WSL cells. Furthermore, the virus genome is stable after passage in WSL cells, and high viral titers can be obtained. Thus, the WSL could be a suitable cell line for the future production of LA-ASFV vaccine candidates, although there may be substantial differences dependent on the particular virus isolate, which would need to be tested on an individual basis.

## Figures and Tables

**Table 1:** Single-nucleotide polymorphisms (SNPs) (underlined) between the blood. Macrophage- and WSL-grown viruses that were present in the coding region.

Gene Name	Genomic Position ^1^	Macrophage-Grown	WSL-Grown
MGF 505-2R	31,813 bp	GCC (Ala)	ACC (Thr)
D250R (g5R)	134,861 bp	GCA (Ala)	ACA (Thr)
I329L (k11L)	169,859 bp	ACG (Thr)	ACA (Thr)

^1^ Genomic position is given relative to Ken06.Bus (GenBank accession: KM111295.

## Data Availability

Not applicable.

## Appendix A

**Table A1:** Primers used for PCR amplification for the generation of products for Sanger sequencing.

Primer	Sequence	Genome Location ^1^	ASFV Gene
p1-f	AGAGTGTATCTCCGCGAAACC	31,696	MGF505-2R
p1-r	TACGGCTTGGGAGAGGACG	32,007	
p2-f	GACCGCATGTGGTATCATATTTGGA	134,732	D250R[g5R]
p2-r	TCGGCAATTCGGGTTTCGTAT	135,129	
p3-f	AGGGCTGTTTGCTGTAGATGC	169,736	I329L[k11L]
p3-r	ACTGCTACCCCTTTGTGTTGGT	169,962	
CD2v_f	CTTCAGGAAGACGTAAATATATGG	69,692	EP402R (CD2v)
CD2v_r	TGAAGGCTAGCTTGAAAGGTT	71,053	

^1^ Genomic position is given relative to Ken06.Bus (GenBank accession: KM111295.1).
